# Mr. Charnock on Purgatives in Typhus

**Published:** 1812-10

**Authors:** R. Charnock

**Affiliations:** Cartmel


*To the Editors of the Medical and Physical Journal.*

GENTLEMEN,

SHOULD the following short notices, concerning the utility of purgatives in the cure of Typhus, appear worthy a place in your widely-extended publication, the insertion of them will oblige

Your most obedient and humble Servant,

*Cartmel, Aug.* 4, 1812. R. CHARNOCK.

Typhus, in the progress of medical science, has been the subject of different modes of practice; but, in modern times, those resulting from the systems of Cullen and Brown have been the most prevalent. Unfortunately, however, the excessive stimulant plan, so strongly recommended in the specious system of the latter ingenious author, has been seldom applied with much advantage by its abettors; nor have the efforts of the Cullenians been more availing. The frequent disappointments in the cure of this disease, therefore, induced me lately to try the effect of purgatives, as recommended by Dr. Hamilton, and further corroborated by the excellent remarks of Dr. Pigot, in a former Number of your Journal; and, from several cases in which I have employed the same successfully, I think I am fully warranted to say, that it is a mode of practice, which will often arrest the progress of Typhus much sooner than any other I have seen adopted.

The purgatives which I have commonly used, were calomel and jalap, combined in the proportions of from vj to x grains of the former, to from ℈j to ℈iifs of the latter, every morning, unless a diarrhœa occurred spontaneously. In most cases, if the medicines were omitted a single day, there was a return of unfavorable symptoms. I have not seen any material advantage derived from the use of purgatives, unless nine, ten, or more motions were produced in the course of twenty-four hours.

Though mercury was freely administered in all cases, as above stated, and one patient took two drachms of calomel and six drachms of jalap in the course of twelve days, yet I never observed the least tendency to ptyalism, excited by the quantity.

In no case, where I have carried this plan to its fullest extent at the beginning of the disease, were the patients ever delirious; nor have I seen that anasarcous tendency occur in the latter stages of the complaint, as is common when the old practice is adopted.

From the success of this mode of treatment, I think we may infer, that any degree of debility, which is induced by

5 emptying emptying the primæ viæ of such offending matter, as constantly attends and modifies the symptoms of Typhus, is only transient; and that we may often, by a salutary stimulus, in this manner, prevent the disease from continuing its baneful influence over the constitution, which soon regains its strength and vigor.

But, though I have treated several cases successfully in this manner, yet I would by no means have practitioners to conclude, that I consider the practice as likely to be of the same utility in all cases of Typhus. Every person must be sensible, that different modes of treatment will be requisite, according to the state of the constitution, and various concomitant circumstances which modify the disease. Hence, in my practice, when the action of the arterial system has seemed violent, and there was considerable determination to the head, I have not hesitated to open the temporal artery, and take away sixteen or twenty ounces of blood; for, in the generality of cases which I have seen, a state of debility did not occur, until the system was reduced, by a long continuance of the fever.

